# Quality of life in elderly adults before and after hearing aid fitting

**DOI:** 10.1590/S1808-86942012000300010

**Published:** 2015-10-14

**Authors:** Maria Fernanda Capoani Garcia Mondelli, Patrícia Jorge Soalheiro de Souza

**Affiliations:** a PhD (Professor – Dental School of Bauru-USP).; b Graduated from the Dental School of Bauru - USP. (Speech and Hearing Therapist). Dental School of Bauru- University of São Paulo.

**Keywords:** hearing aids, hearing loss, quality of life

## Abstract

Presbycusis is a common disorder in the elderly, which causes hearing loss and may contribute to the development of some psychiatric disorders, leading to isolation due to communication difficulties in the social environment.

**Objective:**

To identify through the WHOQOL (World Health Organization Quality of Life Questionnaire), the quality of life of hearing impaired individuals before and after hearing aid fittings.

**Method:**

We had 30 individuals with hearing loss, all over 60 years of age - patients from a Speech Therapy Clinic. The patients answered the WHOQOL questions without the use of hearing aids; and after the effective use of a sound amplification device for a period of three months they answered it again. The WHOQOL - Bref consists of 26 questions, two general quality-of-life questions and 24 associated with four aspects: physical, psychological, environmental and social relations.

**Results:**

There was a significant improvement in quality of life in general, as far as leisure activities were concerned, there were no major changes regarding the frequency of negative feelings; even after the hearing aid fitting, the patients continue to have such feelings.

**Conclusion:**

The use of hearing aids favored the overall quality of life of the individuals evaluated.

## INTRODUCTION

There are changes which make it difficult for the individual to adapt himself to the environment, exactly because of the lack of conditions which favor biopsychosocial aging. The individual's appearance changes, enabling one to give him/her an age estimate with very little margin for error. The skin wrinkles in consequence of dehydration and loss of elasticity of the underlying dermal tissue. Teeth are lost, there are muscle atrophy and joint sclerosis causing movement disorders. Osteoporosis plagues the skeleton and renders it subject to fractures. The heart changes in function, the organs of the senses are impaired^1^.

The elderly are more vulnerable to insidious degenerative diseases, such as cardiovascular and cerebrovascular, cancer, mental and other disorders, which affect the locomotion system and the organs of the senses. Undeniably, there is a systematic reduction in the degree of social interaction as one of the most evident signs of aging^1^.

Hearing loss is one of the most devastating sensorial deficiencies, because it compromises communication and causes emotional, social and occupational sequelae. Hearing loss is usually a harbinger of aging^2^.

According to IBGE data (2005), because of demographic transition, Brazil is among the 10 countries with the higher number of elderly, with the forecast that, in 2025, 14% of our population will be over 65 years of age^3^.

Age-related hearing loss may start at any time; being more common in individuals above 60 years^4^. The effects of age on the peripheral and central auditory systems interact with changes in the reduction of cognitive support, reduction in perception and reduction in speech understanding in noise and in reverberating environments^5^.

Presbycusis is one of the most common disorders reported by elderly individuals, and for most of them, descending bilateral sensorineural hearing loss. Speech understanding difficulty^6^ is associated with this change in hearing thresholds.

Besides the hearing limitation stemming from the acquired hearing loss, some problems must be stressed, such as: the hearing loss and the hearing handicap^7^. The former is associated with the lack of ability to perceive speech in noisy environments, television, etc. The latter concerns the non-auditory aspects, which prevent the individual from properly playing its role in society^2^.

Hearing loss in advanced age is a serious limiting factor for the individual; and it may also lead to psychiatric problems, causing those with hearing loss to isolate themselves because of the difficulty in communicating in the social environment where they live. Often times, the family of hearing impaired patients do not have the patience to deal with the hearing problem and, usually, do not keep normal dialogues with the patient, only informing him/her of essential matters. The elderly feel embarrassed because of his/her difficulty in hearing, which may contribute to depression.

Besides physical problems, the elderly also have higher prevalence of depressive symptoms^8^, ^9^, ^10^, ^11^, being more exposed to abuse and negligence by family members and care takers^12^.

As a result of the forecasted aging of the population, the number of patient candidates to fitting an Individual Sound Amplification Device (ISAD) will greatly increase in the next years^13^.

Today, in the current demographic settings, it is urgent to establish the guidelines for the development of diagnostic programs, acquisition of individual sound amplification device and, especially, of a specific hearing reeducation program for the elderly with hearing loss, so that they can participate and enjoy social relations, having a good quality of life^14^.

Quality of life associated with the health and the subjective health status are similar concepts, centered in the patient's assessment, but necessarily connected to the health status impact on the individual's capacity to live fully^15^.

It is an eminently human notion, which has been brought closer to the degree of satisfaction found in family life, love life, social and environmental lives and their own existential esthetics. The term involves numerous meanings, which reflect knowledge, experience and values from individuals and the collection which report to him at different eras, spaces and histories, being, therefore, a social construction with the brand of cultural relativity^16^.

According to the World Health Organization's Quality of Life concept, it is the individual's perception of his/her own projection in life, within the context of culture and value systems in which he lives and in relation to objectives, expectations, patterns and concerns”^17^.

Quality of Life may be assessed by means of a standardized questionnaire by the group which studies quality of life at the World Health Organization, the World Health Organization Quality of Life Questionnaire (WHOQOL - BREF, 1998), which is an abridged version of a longer questionnaire, with 100 questions^18^.

Some authors have stated that, the better a person's adaptation to life at earlier ages, the better will be such adaptation at old age. The elderly with better fitting qualities (personalities) would not manifest mental disorders when facing similar life conditions. One of the most important psychological aspects when one talks about old age concerns exactly the ability of people to adapt; thus, old age means a great challenge to contemporary humans^19^.

Thus, with the increase in the number of elderly citizens with hearing loss, and their consequent isolation, the goal of the present paper is to use WHOQOL (World Health Organization Quality of Life Questionnaire) to assess the individual's quality of life before and after fitting a hearing aid (ISAD).

## METHOD

This study was carried out with the approval of the Ethics in Research Committee, under protocol #002/2009. All participants in this study consented with the publication of its results.

The study was made up by 30 individuals with a mean age of 76.8 years, from both genders, seen at the Department of Selection and Fitting of Hearing Aids in the period between August, 2009; and May of 2010.

Inclusion criteria:

• Signature in the Free and Informed Consent Form.
• Age range between 60 and 90 years.
• Diagnosis of bilateral moderate sensorineural hearing loss.
• Good general health status, starting from the assumption that if the patient was able to go the clinic for care; he/she would also be able to participate in the study.
• Without experience concerning the use of ISAD on the first use of the questionnaire.
• Have understanding capacity to answer the WHOQOL-Bref questionnaire.
• Effective use of the ISAD (daily for more than 6 hours) for three months for the second questionnaire.
• Come to the return visits schedule by the service. Exclusion criteria:
• No depression reported by the individual during the psychological interview and at the time of the study according to criteria from the institution's psychologist. The test adopted was the Beck's Depression Inventory - (BDI-II).
• Hearing loss preventing the questionnaire's understanding.

After proper audiologic diagnosis, the patient answered the questions of the WHOQOL - Bref^20^ questionnaire, which were asked by the researcher (assisted deployment). The patients were not given synonyms or were explained the question in other words - to avoid changing its original meaning. When the respondent was unable to read the questionnaire because of health issues or literacy problems, the questionnaire was read by the interviewer (managed deployment), in order to assess the quality of life level of the hearing impaired without the sound amplification.

We selected and fit the sound amplification devices according to the need of each subject; and each patient was advised and educated concerning the use and cleaning of their devices.

The patients returned monthly to check for the need of doing fine adjustments in the programming of their hearing aids, and clarification of doubts, thus guaranteeing proper use of the hearing aids. It is worth stressing that there was no hearing training during this period.

The elderly made an effective use of the device for three months and, after this period, answered the questions again (the questionnaire was again applied by the researcher), with the aim of checking whether the use of amplification provided for a quality of life improvement.

The effectiveness concerning the ISAD use was considered when the individual reported its daily use for longer than six hours/day during the three months considered for employing the WHOQOL - Bref questionnaire.

The WHOQOL - Bref is made up of 26 questions, having two general questions, associated with quality of life and 24 representing each one of the 24 aspects which make up the original instrument. The information from which this abridged version stemmed was taken from field tests done in 20 centers in 18 different countries. This questionnaire cropped up from the need for short questionnaires which require less time to answer; however with reasonable psychometric characteristics^18^. It sets four aspects apart: physical, psychological, environment and social relations.

The WHOQOL-Bref bears the following demand:

1. Physical: corresponds to questions associated with pain, discomfort, energy, fatigue, sleep, rest, mobility, daily life activity, medication or treatment dependence and work capacity.
2. Psychological: encompasses issues about positive feelings, thinking, learning, memory and concentration, self-esteem, body image and appearance, negative feelings, spirituality, religion and personal beliefs.
3. Environment: involves physical safety and protection, home environment, financial resources, health and social care: availability and quality, opportunities to acquire information and skills; participation in and opportunities for recreation/ leisure, physical environment (pollution/noise/ traffic/climate) and transportation.
4. Social relations: questions about personal relations, social support and sexual activity. Individuals answered based on gradations, such as: very bad, bad, neither bad nor good, good and very good or very unhappy, unhappy, neither unhappy nor happy, happy, very happy, depending on the aspect investigated the questionnaire offers these options.

After filling out the questionnaire, we calculated the general values and those by aspect (physical, psychological, environmental and social relations), enabling an assessment of the individual's quality of life. This analysis was carried out according to the syntaxes proposed by the translators, with the help of the Statistical Package for Social Science (SPSS) 10.0 for Windows, when counting and manual analysis was advised against because of a greater likelihood of errors^21^.

## RESULTS

The results consider the 30 participants who returned in the three-month period and answered all the questions in the questionnaire - 17 men (56.6%) and 13 women (43.4%).

**Graph 1:**
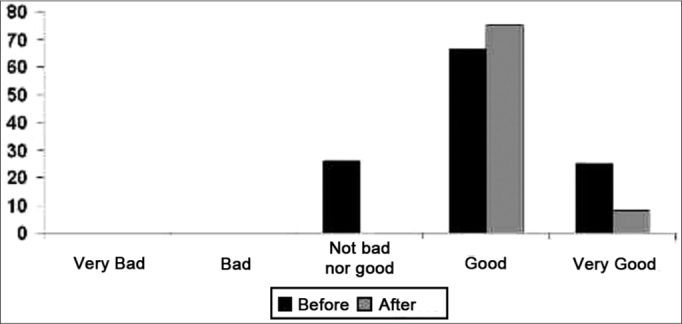
Results of the general values concerning the quality of life of elderly.

After comparing the results from the WHOQOL questionnaire, answered before and after fitting a hearing aid, one can notice a significant improvement in quality of life in general, since, after its fitting, all the patients ranked their quality of life as good or very good. Graph 1 depicts the answers obtained from the patients and the general result of the assessment, and the maximum score with the ISAD was equal to 75, in the 0-100 point-scale.

**Graph 2:**
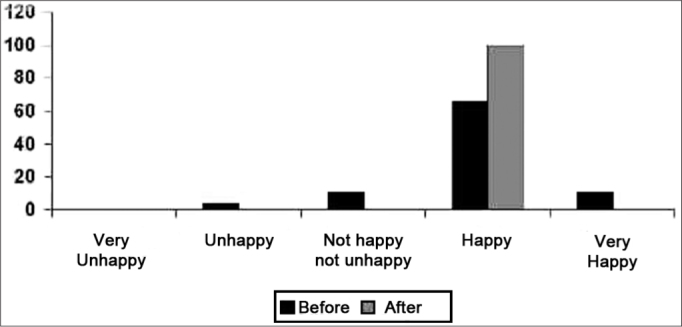
Results from the answers concerning physical aspects.

As far as health is concerned, after fitting the hearing aid, all patients seemed happy according to the questionnaire, suggesting that for some patients who were hearing challenged before the hearing aid, it was associated with a health problem(Graph 2).

**Graph 3:**
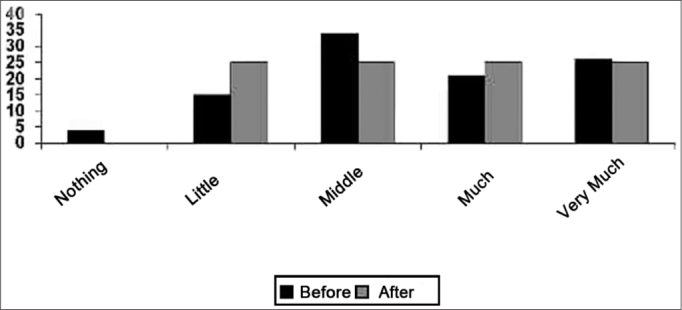
Results from the answers concerning psychological aspects.

As to leisure opportunities, there were no major changes, given that the answers to the questionnaire given afterwards were well split among the patients, and very similar to the answers found in the pre-fitting questionnaire (Graph 3).

**Graph 4:**
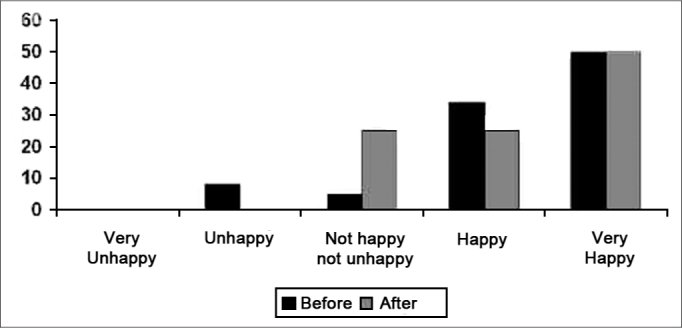
Results from the answers concerning environment aspects.

When asked about satisfaction with personal relations, between the patient and friends, relatives, colleagues and acquaintances, half of the participants remained very pleased. The other half, were less happy when the pre fitting situation was compared to the post-fitting one; conversely, the mean response increased, and there were no answers of unhappiness.(Graph 4).

As to the frequency of negative feelings, we noticed that, even after fitting the hearing aid, the patients continued having such feelings, even when they were not often or frequent. This is due to the fact that loosing hearing acuity is not the only reason why the elderly feel depression, anxiety and other noxious emotions (Graph 5).

**Chart 5:**
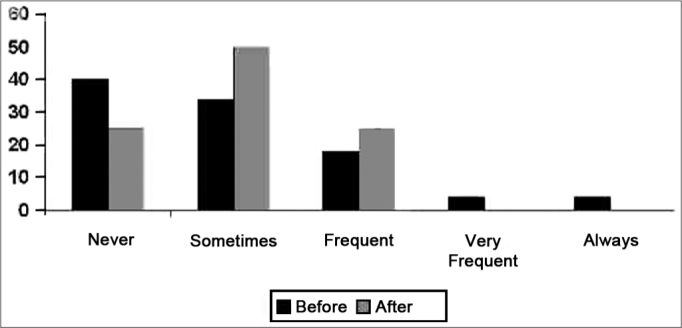
Results from the answers concerning social relations aspects.

## DISCUSSION

In today's world, the elderly are seen by our society as disabled people, without a social role to play, compromising productivity since it stresses youth vitality. Modern society is exclusively founded on productivity and, considering social functions, youth is seen as being better. Thus, the elderly feel disappointed with losing their roles, previously lived in full and successfully, causing social isolation and privation from the sources of communication and information, responsible for keeping the individual active in society^22^.

Some authors stated that, the better the person's adaptation to life in earlier ages, the better the person will adapt him/herself in older ages. Elderly who are better able to cope (personality), did not have emotional disorders when having to face similar life situations. One of the most important psychological aspects of older age is associated to the person's capacity to cope. Thus, growing old becomes a major challenge for our contemporary society^19^.

Having all these challenges, the elderly still have to face a difficulty to communicate with others, stemming from hearing loss resulting from aging itself, compromising their relationships with family and friends, one more impact to their psychosocial lives^22^.

The isolation of the elderly and the consequent drop in the quality of their communication, because of sensorial deficits, have a profound impact, since it is the constant flow of communication and information that keeps the individual active in society^23^.

To be a senior citizen with acquired hearing loss is something which goes beyond the hearing impairment itself, causing severe psychosocial implications for his life and that of those who live with him^24^.

Poor social relations are a health risk factor, and it has been considered as harmful as smoking, high blood pressure, obesity and the lack of physical activities^25^.

Early diagnosis and treatment of age-related hearing loss are fundamental for a good quality of life for senior citizens. Studies and research in this field point to the possibility of a functional change based on brain plasticity, even when considering adult individuals^26^.

Results have shown that, after using ISAD, there is improvement in the individuals quality of life as a whole (Graph 1), stressing the importance of amplification and referral of users to fitting programs and training in communication strategies^27^, ^28^, ^29^, ^30^.

In the physical realm, there was a significant difference, with score improvements, and one can see a global improvement when comparing pre and post-fitting data. Such data differ from those in the study carried out in Porto Alegre^31^.

The psychological aspect improved with the ISAD, stressing the importance of hearing for an individual's general quality of life and health^32^.

As far as the environment is concerned, there was no statistically significant difference. The questions associated with it encompass physical safety, home environment, financial resources; healthcare and social (availability and quality); skills, information opportunities, recreation and leisure; physical environment (pollution, noise, traffic, climate) and transportation^18^. Such items are associated with public health, and are not closely related to the effective use of an ISAD. Studies have stressed that environmental factors, such as basic sanitation, public safety, social and healthcare, pollution, traffic, transportation and the climate have a negative impact on the QL of the Brazilian population^33^, ^34^, ^35^.

There was a significant improvement as far as social relations are concerned. It is believed that those individuals who participated more actively in the groups, had a more active participation in society avoided social isolation. More than one month to have the questionnaires answered again may have favored the results^31^, because of aclimatization^36^.

Moreover, it is necessary to create or implement programs aiming at reintegrating the individual to society, especially the elderly. One alternative would be to refer these individuals to group meetings, in order to improve their social relations.

Together with another study^14^, we may suggest that public healthcare services, involving physicians and speech and hearing therapists, must establish the guidelines concerning the development of diagnostic programs, ISAD acquisition and, especially, hearing reeducation for senior citizens with hearing loss, so that they can enjoy their social lives, keeping a good quality of life.

The primary tool used in the rehabilitation of people with hearing loss is the individual sound amplification device; however, there is also the traditional hearing rehabilitation, which includes hearing training and instructions for speech understanding^37^.

In this study, the patients followed the same criteria of the other patients seen in the clinic, with monthly return visits to have questions answered and go through the necessary adjustments in their ISAD. Hearing training was not given. Communication strategies are part of the post-fitting instructions and are given to all patients equally; thus, they were not considered to be discussed in the present study.

## CONCLUSION

This study showed that using an ISAD improved the overall quality of life of the individuals assessed.

## Figures and Tables

**Table:** 

	Nothing	Very Little	average	Much	Plenty
Do you get from others the support you need?	1	2	3	4	5

**Table:** 

	Nothing	Very little	Average	Much	Plenty
Do you get from others the support you need?	1	2	3	4	5

You must circle number 1 if you received no support at all.

**Table:** 

1. How would you rate your quality of life?	Very bad	Bad	Not bad, and not good	Good	Very good

1	2	3	4	5

2. How happy are you with your health	Very happy	Unhappy	Not happy, and not unhappy	Happy	Very happy
	1	2	3	4	5

**Table:** 

	Nothing	Very little	More or less	Much	Very Much
3. To what extent do you think your pain (physical) prevents you from doing what you need?	1	2	3	4	5

4. To what extent do you need some medical treatment to live your daily life?	1	2	3	4	5

5. How much do you enjoy life?	1	2	3	4	5

6. How much do you think your life makes sense?	1	2	3	4	5

7. How much can you concentrate?	1	2	3	4	5

8. How safe do you feel in your daily life?	1	2	3	4	5

9. How healthy is your physical environment (climate, noise, pollution, attractions)?	1	2	3	4	5

**Table:** 

	Nothing	Very little	Medium	Much	Fully
10. Do you have enough energy for your daily life?	1	2	3	4	5

11. Are you able to accept your physical appearance?	1	2	3	4	5

12. Do you have enough money to pay for your needs?	1	2	3	4	5

13. How available for you is the information you need for your daily life?	1	2	3	4	5

14. To what extent do you have leisure activities?	1	2	3	4	5

**Table:** 

15. How well can you move?	Very bad	Bad	Not bad and not good	Good	Very good

1	2	3	4	5

	Very unhappy	Unhappy	Not happy but not unhappy	Happy	Very happy

16. How happy are you with your sleep	1	2	3	4	5

17. How happy are you with your capacity to perform your daily activities?	1	2	3	4	5

18. How happy are you with your capacity to work?	1	2	3	4	5

19. How happy are you with yourself?	1	2	3	4	5

20. How happy are you with your personal relations (friends, relatives, acquaintances and colleagues)?	1	2	3	4	5

21. How happy are you with your sex life?	1	2	3	4	5

22. How happy are you with the support your get from your friends?	1	2	3	4	5

23. How happy are you with the life conditions where you live?	1	2	3	4	5

24. How happy are you with your access to healthcare?	1	2	3	4	5

25. How happy are you with your means of transportation?	1	2	3	4	5

**Table:** 

	Never

26. How frequent do you have negative feelings, such as bad mood, hopelessness, anxiety, depression?	

## Attachment 1 Instructions

This questionnaire is about how you feel regarding your quality of life, health and other aspects of your life. **Please, answer all the questions.** If you are not sure about how to answer a question, please choose the alternative which feels the most appropriate for you. Often times, this may be your first choice. Please, have in mind your values, aspirations, likings and concerns. We are asking about what you think of your life, considering the **last two weeks.** For instance, considering the last two weeks, one question could be:

You should circle the number which best corresponds to how much support you get from others for your needs in these last two weeks. Therefore, you must circle the number 4 if you received “much” support as shown below.

Please, read each question, think about it and circle the number which seems to be the best answer.

The following questions are about **how much you have felt** some things in the past two weeks.

The following questions investigate how fully you have felt or were able to do certain things in these past two weeks.

The following questions investigate how well or happy you have felt concerning different aspects of your life in the past two weeks.

The following questions are associated with how frequent you felt or experienced certain things in the past two weeks.

**THANK YOU FOR YOUR HELP.**
